# Early Adherence to Immunomodulators in Multiple Myeloma: Retrospective Observations from a Safety-Net Hospital

**DOI:** 10.3390/curroncol33070432

**Published:** 2026-07-19

**Authors:** Onyebuchi Ononogbu, Sydney Mohr, Javeria Khalid, Alyssa Glass, Jane Emovon, Hilary Ma, Yaser Alkhatib, Amit Correa

**Affiliations:** 1 Department of Pharmacy Practice and Translational Research, College of Pharmacy, University of Houston, Houston, TX 77204, USA; 2 Department of Pharmaceutical Health Outcomes and Policy, College of Pharmacy, University of Houston, Houston, TX 77204, USA; 3 Department of General Oncology, The University of Texas MD Anderson Cancer Center, Houston, TX 77030, USA; 4 Lyndon B. Johonson Outpatient Hematology, Harris Health System, Houston, TX 77030, USA; 5 Department of Medical Oncology, Cleveland Clinic Abu Dhabi, Abu Dhabi 112412, United Arab Emirates; 6 McGovern Medical School, The University of Texas Health Science Center at Houston, Houston, TX 77030, USA

**Keywords:** multiple myeloma, immunomodulators, medication adherence, proportion of days covered, safety-net hospital, health disparities, Patient Medication Assistance Program, REMS, lenalidomide, pomalidomide

## Abstract

**Background/Objectives:** Multiple myeloma (MM) disproportionately affects older adults and racial/ethnic minorities, many of whom face socioeconomic barriers to care. Immunomodulators (IMiDs) are central to MM therapy, but their benefit relies on timely initiation and sustained adherence. In safety-net settings, barriers such as insurance approvals, Patient Medication Assistance Program (PMAP) enrollment, and Risk Evaluation and Mitigation Strategy (REMS) requirements may influence adherence. This study evaluated IMiD adherence during the first two years of therapy among socioeconomically vulnerable MM patients in a large safety-net hospital, with attention to PMAP enrollment and treatment delays. **Methods:** We conducted a retrospective cohort study of adults (≥18 years) with newly diagnosed MM between October 2010 and January 2022 who initiated lenalidomide or pomalidomide at a safety-net hospital. Adherence was measured using the proportion of days covered (PDC). Delays in initiation were defined as ≥1 month from diagnosis to first IMiD prescription fill. This threshold was selected a priori based on the REMS-mandated 28-day prescription supply limit for lenalidomide and pomalidomide, which establishes a natural monthly treatment cycle; a delay exceeding one full 28-day cycle before therapy initiation is operationally meaningful within the REMS framework. **Results:** Eighty patients met criteria (mean age 55.1 years; 56.3% Hispanic/Latino; 31.3% African American). Most were unemployed (63.8%), 42.5% uninsured, and 57.5% enrolled in PMAP. Only 12.5% achieved PDC ≥ 80%. Median PDC was higher among PMAP enrollees (0.40 vs. 0.31; *p* = 0.0293) and transplant-ineligible patients (0.46 vs. 0.27; *p* = 0.0218). Delays ≥ 1 month occurred in 65% of patients. **Conclusions:** Adherence to IMiDs was suboptimal in this younger safety-net cohort. Although PMAP improved adherence, program delays may reduce early treatment benefits, underscoring the need for streamlined access and targeted interventions to address both patient- and system-level barriers.

## Simple Summary

Multiple myeloma is a blood cancer that disproportionately affects racial and ethnic minorities and people with limited financial resources. Immunomodulators, a key drug class for this cancer, work best when taken consistently and started promptly after diagnosis. This study examined medication adherence among 80 patients at a safety-net hospital in Houston, Texas, over two years. Patients were predominantly Hispanic/Latino (56%) and African American (31%), with an average age of 55, and most faced significant financial hardship. Only 1 in 8 patients took their medication consistently enough to be considered adherent. Enrollment in a financial assistance program was associated with better medication possession, highlighting its critical role in sustaining access. However, 65% of patients experienced delays of one month or more before starting treatment, largely due to administrative and regulatory requirements. Streamlining assistance programs and tailoring support for working-age patients with competing responsibilities could meaningfully improve outcomes for this underserved population.

## 1. Introduction

Safety-net hospitals are institutions mandated to provide care regardless of patients’ ability to pay, serving a disproportionately high share of uninsured, underinsured, Medicaid-enrolled, and socioeconomically vulnerable patients [1]. These institutions play a critical role in cancer care delivery for patients who face financial hardship, limited transportation, language barriers, unstable employment, and fragmented access to specialty care [2]. Access to high-cost oral oncology medications in safety-net settings often depends on manufacturer-sponsored assistance programs, insurance authorization, and additional administrative coordination. Unlike academic medical centers, where most prior multiple myeloma adherence research has been conducted, safety-net settings face unique structural challenges, including administrative burden from programs such as the Patient Medication Assistance Program (PMAP) that distinguish them from better-resourced settings. The intersection of disease complexity, polypharmacy, and socioeconomic disadvantage creates a compounding adherence burden that is poorly captured in existing literature, which has predominantly enrolled insured, older, and non-Hispanic White patients [2]. Understanding the structural and patient-level contributors to oral oncology adherence in safety-net settings is therefore essential for designing more equitable cancer care systems.

At our institution, the Patient Medication Assistance Program (PMAP) supports access to high-cost medications for financially vulnerable patients [3]. For IMiDs, PMAP staff coordinate documentation required for manufacturer-sponsored assistance programs (e.g., Bristol Myers Squibb Access Support for lenalidomide and pomalidomide), verify patient eligibility, communicate with prescribers and pharmacies, and help ensure completion of REMS-related requirements. Although PMAP may improve sustained access by reducing financial barriers after approval, the enrollment process can delay treatment initiation because medication cannot be dispensed until the required documentation, manufacturer approval, REMS authorization, and pharmacy processing steps are completed.

Multiple myeloma (MM) is a plasma cell malignancy characterized by the accumulation of abnormal plasma cells in the bone marrow, resulting in progressive bone destruction and hematopoietic failure [4]. Diagnostic criteria include ≥10% bone marrow plasma cells or biopsy-proven plasmacytoma, along with one or more myeloma-defining events, including hypercalcemia, renal dysfunction, anemia, lytic bone lesions, or biomarkers of malignancy [5]. Immunomodulators (IMiDs) represent a cornerstone in the treatment of MM due to their dual mechanism of action: direct anti-myeloma cytotoxicity and immune system modulation [6]. Clinical trials demonstrate that IMiDs, often combined with proteasome inhibitors (PIs), steroids, and monoclonal antibodies, improve response rates, progression-free survival (PFS), and overall survival (OS) [1,4].

However, the effectiveness of IMiD therapy depends heavily on consistent adherence. Adherence is particularly challenging in socioeconomically vulnerable populations, who often face barriers such as limited healthcare access, high out-of-pocket costs, housing instability, food insecurity, transportation difficulties, and lower health literacy [7,8]. These challenges are further complicated in MM, which disproportionately affects older adults and racial/ethnic minorities [9]. Notably, our safety-net hospital cohort is younger (mean age 55) than the typical MM population reported in the literature (60s–70s). Younger patients may still be employed and caring for families, which could add competing priorities that influence adherence in ways not commonly described in previous studies. Research in adjacent oral oncology settings—including endocrine therapy for breast cancer—has demonstrated that working-age patients with low socioeconomic status exhibit lower adherence due to employment instability, inadequate insurance coverage, and difficulty navigating complex prescription assistance pathways [8]. These dynamics are likely amplified in MM, where REMS requirements add an additional administrative layer demanding consistent patient engagement every 28 days regardless of competing life demands.

Although progression-free survival has improved substantially with modern multiple myeloma therapy, timely treatment initiation and sustained adherence remain critical to optimizing disease control and reducing avoidable gaps in care [10,11,12]. While prior studies demonstrate lower adherence among low-SES populations [7,8,13], limited data exist on how systemic barriers within safety-net hospitals—such as those related to medication access programs or regulatory requirements—affect adherence patterns. The racial and ethnic composition of safety-net MM cohorts is also distinct: African American patients, who are disproportionately represented in safety-net settings, are diagnosed at younger ages and with more aggressive disease characteristics, yet remain underrepresented in clinical trials and adherence research [9]. Closing this gap requires studies conducted specifically within safety-net systems, where the structural and demographic realities of underserved populations can be examined with appropriate granularity [7,8,13]. Limited data exist on how systemic barriers within safety-net hospitals, such as those related to medication access programs or regulatory requirements, affect adherence patterns.

The objective of this study was to evaluate adherence to IMiD therapy during the first two years of treatment among socioeconomically vulnerable MM patients treated at a safety-net hospital, with particular focus on the impact of the PMAP and treatment delays on adherence.

## 2. Materials and Methods

### 2.1. Patients

This retrospective, observational study evaluated medication adherence during the first two years of IMiD use among newly diagnosed multiple myeloma patients treated at Harris Health System, a safety-net hospital, in Houston, Texas. This study was conducted under protocols approved by the appropriate institutional review boards. Eligible patients were ≥18 years of age, had a new diagnosis of multiple myeloma between October 2010 and January 2022, and were prescribed lenalidomide or pomalidomide as IMiD therapy. Patients without evidence of an IMiD regimen, those receiving care outside of the safety-net hospital system, and those treated with thalidomide were excluded, as thalidomide is not on formulary at our institution and was not routinely available through the hospital medication access pathway during the study period. A total of 124 patients were identified through data extraction, 113 charts were retrospectively reviewed, and 80 met full inclusion criteria.

### 2.2. Data Collection

Patient medical record numbers for individuals prescribed lenalidomide or pomalidomide were extracted from the electronic health record (EHR) to generate the study cohort. Comprehensive chart review was conducted to verify IMiD prescription and adherence data and to collect demographic and clinical characteristics, including race/ethnicity, sex, body mass index (BMI), insurance status, comorbidities, and treatment regimen. Employment status was categorized as employed, unemployed, or other; the ‘other’ category captured patients documented as retired, disabled, or with undocumented employment status, as the medical record did not consistently distinguish between these groups.

Eligible patients received IMiD-containing regimens with lenalidomide or pomalidomide. Patients may have received IMiDs as part of combination regimens that included corticosteroids, proteasome inhibitors, monoclonal antibodies, or other anti-myeloma therapies, depending on treatment era, disease characteristics, transplant eligibility, and clinician preference. The adherence analysis focused specifically on the IMiD component of therapy, as these oral agents are dispensed through the outpatient pharmacy under REMS and are amenable to PDC-based analysis. IMiDs are subject to the U.S. Food and Drug Administration’s (FDA) Risk Evaluation and Mitigation Strategy (REMS) program due to their potential for serious adverse effects. The REMS program requires that IMiD prescriptions be dispensed in a maximum 28-day supply with no automatic refills [13]. Each cycle, patients must complete required laboratory assessments and documentation before the next prescription can be dispensed.

At our institution, the Patient Medication Assistance Program (PMAP) serves as an intermediary between patients, prescribers, and manufacturer-sponsored assistance programs (e.g., Bristol Myers Squibb Access Support for lenalidomide and pomalidomide) to facilitate medication access for uninsured and underinsured patients. The PMAP enrollment process involves sequential steps: patient eligibility screening, application submission to the manufacturer, manufacturer review and approval, and coordination of medication dispensing. Because medication cannot be dispensed until manufacturer approval is secured, patients who depend on PMAP may experience a delay between diagnosis and first prescription fill. Once approved, PMAP provides consistent monthly medication access, which likely explains the higher long-term PDC observed among enrolled patients.

### 2.3. Adherence Measurement

Medication adherence was assessed using the proportion of days covered (PDC), calculated by dividing the total days’ supply dispensed during the 24-month observation period by the total number of days in the observation window [14]. PDC was summarized as a continuous variable and dichotomized using a threshold of ≥80% to classify patients as adherent or non-adherent, consistent with established benchmarks in oral oncology adherence research. Missed prescription fills or gaps in medication possession contributed to lower cumulative PDC values. PDC reflects medication possession rather than confirmed ingestion and, in the context of a safety-net health system, may capture both patient-level adherence behaviors and systemic barriers such as insurance authorization delays or PMAP processing requirements [13]. The date of the first IMiD prescription fill served as the index date for the 24-month adherence observation period. The date of MM diagnosis was used solely to calculate time to treatment initiation and was not used as the start of the adherence observation period.

A treatment initiation delay of ≥1 month was defined a priori based on the REMS-mandated 28-day prescription supply limit for lenalidomide and pomalidomide, which establishes a natural monthly treatment cycle. A delay exceeding one full 28-day cycle before therapy initiation was considered clinically and operationally meaningful within the REMS-governed dispensing framework.

### 2.4. Statistical Analysis

Medication adherence was summarized using PDC as the primary metric. The primary endpoint was the median PDC over the 24-month follow-up. Descriptive statistics (means or medians for continuous variables, percentages for categorical variables) were used to characterize the cohort. Differences in PDC between subgroups were assessed using independent-sample *t*-tests for normally distributed variables and Kruskal–Wallis tests for non-normal distributions. All analyses were conducted using SAS version 9.4 (SAS Institute, Cary, NC, USA).

## 3. Results

Eighty patients met the inclusion criteria and were analyzed. The mean age at diagnosis was 55.1 years (SD ± 9.0; median 55 years; range 37–81 years), which is notably younger than typical multiple myeloma populations reported in the literature. The cohort was predominantly Hispanic/Latino (56.3%) and African American (31.3%), with a slight female predominance (55.0%). Most patients were unemployed or homeless (63.8%), and 42.5% were uninsured, with 57.5% enrolled in PMAP. Over a third of patients were obese (33.8%). By ISS stage, the majority were Stage 2 (43.8%), while 22.5% were Stage 3 and 17.5% were unstaged. Cytogenetic high-risk disease was identified in 30.0% of patients, and ECOG performance status was ≤2 in 82.5%. Bone disease was present in the large majority of patients (86.3%). The most prevalent comorbidities were depression (40.0%) and hypertension (35.0%). Most patients received a standard VRD induction regimen (70.0%), nearly all were prescribed lenalidomide (95.0%), and 53.8% were transplant-ineligible. At last follow-up, 77.5% of patients were alive. Baseline demographic and clinical characteristics are presented in Table 1.

Bivariate analysis of PDC by patient subgroup is presented in Table 2. The overall mean PDC was 0.42 ± 0.25, and the median PDC was 0.347. Only 10 patients (12.5%) achieved the ≥80% PDC threshold associated with improved clinical outcomes, while the remaining 70 patients (87.5%) fell below this benchmark. PMAP enrollment was significantly associated with higher median PDC (0.40 vs. 0.31; *p* = 0.029), and transplant-ineligible patients had significantly higher median PDC than transplant-eligible patients (0.46 vs. 0.27; *p* = 0.022). No statistically significant differences in PDC were observed across other subgroups, including sex, race/ethnicity, employment status, insurance type, ISS stage, or treatment regimen. Delays in treatment initiation, defined as a gap of at least one month from diagnosis to the first prescription refill, were observed in 65% of patients (*n* = 52). These patients had a higher median PDC compared to those without a delay (0.444 vs. 0.33), though the difference was not statistically significant (*p* = 0.413). The median time from MM diagnosis to first IMiD prescription fill was 35 days (range 0–1035 days), reflecting substantial heterogeneity in treatment initiation timelines across this cohort.

## 4. Discussion

This study examined adherence to immunomodulators (IMiDs) during the first two years of therapy in a socioeconomically vulnerable, racially and ethnically diverse MM population treated within a safety-net hospital system. Overall, adherence rates were low, with only 12.5% of patients meeting the ≥80% PDC benchmark typically associated with improved outcomes in MM and other chronic conditions. These findings are consistent with prior studies showing that patients with lower socioeconomic status (SES) face greater barriers to maintaining oral oncology adherence [7,8,13].

Our cohort was notably younger than populations typically reported in MM studies (mean age 55 versus 60s–70s). Many of these patients may still be actively employed and carrying family or caregiving responsibilities. These competing demands, alongside financial stressors, could further complicate adherence [15]. Although our quantitative analysis did not identify age as a statistically significant factor, the socioeconomic realities of a younger cohort may intersect with adherence in ways not captured by PDC metrics. Future work should explore how employment, caregiving, and household income dynamics shape oral oncology adherence among younger MM patients. The younger mean age of our cohort (55.1 years; range 37–81) likely reflects the demographic characteristics of the safety-net population served by our institution, including a high proportion of uninsured and underinsured working-age adults who may present with symptomatic MM at younger ages due to limited primary care access. The predominantly Hispanic/Latino and African American composition of our cohort may also contribute, as prior literature has documented earlier MM onset among African American patients. These findings are therefore most applicable to working-age, socioeconomically vulnerable patients in safety-net settings.

The PDC metric used here reflects both patient- and system-level barriers. While PDC is widely accepted as a proxy for adherence, in the safety-net context it is inherently influenced by systemic processes such as insurance approvals, REMS restrictions, and PMAP enrollment timelines. Thus, lower PDC may not solely represent patient nonadherence but also structural barriers to timely medication access. This dual signal complicates interpretation and underscores the need for complementary qualitative studies to disentangle patient-driven versus system-driven adherence challenges. Patient-level barriers in this cohort included financial hardship (42.5% uninsured), competing employment and caregiving responsibilities, transportation difficulties, and the monthly laboratory and documentation requirements imposed by REMS. System-level barriers included PMAP enrollment processing timelines, insurance authorization delays, and REMS-mandated 28-day supply limits with no automatic refills. This heterogeneity in the drivers of low PDC has important methodological implications: future studies in safety-net settings should incorporate prospective documentation of the reason for each missed fill—distinguishing patient behavior from system failure—rather than relying solely on dispensing records. Pharmacy-based interventions, including proactive refill outreach, adherence dashboards, and pharmacist-led counseling, have shown promise in improving oral oncology adherence in other settings and warrant prospective evaluation in safety-net populations [16].

PMAP enrollment was associated with higher median PDC, reinforcing the critical role of financial support in sustaining treatment. For patients who would otherwise be unable to afford high-cost oral agents, PMAP is practically the only route to access therapy. While enrollment improved medication possession, it frequently delayed therapy initiation, as medication could not be dispensed until program approval. This paradox—improved long-term adherence but delayed therapy initiation—represents a previously underreported dynamic with direct implications for early disease control in MM, where disease activity in the first months after diagnosis can significantly influence long-term outcomes [10]. Manufacturer-sponsored patient assistance programs have been shown to reduce financial toxicity and support medication continuity in oncology settings, though their administrative complexity can paradoxically delay the very access they are designed to provide [17]. Process optimization within PMAP—including pre-enrollment screening initiated at the time of diagnosis, parallel application workflows, and dedicated PMAP navigation staff—could preserve both timely initiation and sustained long-term adherence, representing a high-yield target for quality improvement.

Treatment initiation delays were common, occurring in 65% of patients—a rate that substantially exceeds those reported in broader, predominantly insured MM populations [18]. While these patients exhibited slightly higher median PDC once therapy was established, the delay itself carries meaningful clinical risk [10]. REMS requirements, which mandate monthly prescriptions and confirmatory labs, likely contribute to administrative complexity and initiation delays. Whether a one-month initiation delay translates into inferior survival outcomes in MM remains an open and clinically important question that warrants prospective evaluation [18,19]. Our documented reasons for delay—PMAP application timelines, insurance authorization, REMS compliance, and medication shipment—reflect a pattern of administrative fragmentation that disproportionately affects uninsured patients. Addressing these delays requires coordinated action across institutions, manufacturers, and pharmacy systems to create smoother pathways from diagnosis to first prescription fill [18,19].

Transplant eligibility also appeared to influence adherence, with transplant-ineligible patients achieving higher PDC. This likely reflects regimen planning: IMiDs remain a long-term mainstay for ineligible patients, whereas transplant-eligible patients often discontinue lenalidomide prior to stem cell mobilization due to its known impact on stem cell collection [20]. Thus, lower adherence in the transplant-eligible group may reflect planned clinical interruptions rather than patient-driven nonadherence. Importantly, our PDC calculation did not exclude periods of planned treatment interruption, an acknowledged limitation of dispensing-record-based adherence metrics.

Several limitations warrant acknowledgment. Disease severity measures, including R-ISS stage and baseline bone marrow plasma cell burden, were not uniformly documented across the study period, limiting our ability to fully evaluate the association between disease risk, disease burden, and IMiD adherence. The retrospective design and reliance on dispensing records preclude confirmation of actual medication ingestion, and single-institution data limit generalizability across safety-net systems. Future multicenter prospective studies should incorporate patient-reported adherence barriers, pharmacist-documented reasons for missed fills, and linkage to clinical outcomes—including response rates, disease progression, and survival—to more fully characterize the adherence landscape in this population [21].

Although our findings reinforce known associations between socioeconomic vulnerability and poor adherence [7,13,17], they add value by illustrating how systemic mechanisms within a safety-net hospital—particularly PMAP enrollment and REMS requirements—directly shape medication access patterns in ways that extend beyond individual patient behavior. These findings point to a clear institutional and policy opportunity: embedding adherence support into the safety-net care infrastructure rather than treating it as secondary to treatment decisions. Pharmacist-led adherence programs, patient navigation services, and proactive PMAP enrollment initiated at diagnosis rather than after treatment planning could meaningfully reduce both initiation delays and long-term non-adherence [16]. Equity-centered quality improvement frameworks should prioritize these interventions for younger, working-age, minority patients—an understudied but high-need group in the MM adherence literature [22]. The intersection of race, age, insurance status, and systemic barriers in this cohort reflects a broader pattern of structural inequity in cancer care that requires targeted, sustained institutional commitment to address [7,13,17]. They add value by illustrating how systemic mechanisms within a safety-net hospital, particularly PMAP enrollment and REMS requirements, directly shape access patterns. Our results highlight opportunities to streamline assistance program processes, reduce initiation delays, and design adherence interventions tailored to the needs of younger, working-age minority patients [22].

## 5. Conclusions

Adherence to IMiD therapy was suboptimal in this socioeconomically vulnerable, racially and ethnically diverse MM cohort. Although vital status was available for all 80 patients (17 deaths, 21.3%; median follow-up 4.3 years), the event rate was insufficient for robust survival analysis, and progression-free survival data were not uniformly available due to the retrospective design and treatment transition confounding, with only 12.5% of patients achieving the ≥80% PDC benchmark. PMAP enrollment was significantly associated with improved medication possession, underscoring its essential role in sustaining treatment access for uninsured and underinsured patients. However, treatment initiation delays occurred in 65% of patients, a rate exceeding those reported in broader MM populations, highlighting the paradoxical impact of administrative and regulatory barriers inherent to safety-net settings. These findings call for targeted, system-level interventions—including streamlined PMAP workflows with proactive enrollment at diagnosis, pharmacist-led adherence support embedded within the oncology care team, REMS-integrated dispensing processes that reduce administrative burden on patients, and adherence strategies specifically designed for younger, working-age minority patients in safety-net settings. Future prospective studies should link adherence metrics to clinical outcomes and incorporate patient-reported barriers to develop a fuller understanding of how non-adherence translates into disease progression and survival in this underserved population. Meaningful progress in MM outcomes equity will require not only effective therapies but also institutional commitment to ensure that the patients who need them most can consistently access and sustain them to address the compounding patient- and system-level barriers to oral oncology adherence.

## Figures and Tables

**Table 1:** Baseline demographic and clinical characteristics of multiple myeloma patients at a safety-net hospital (*n* = 80).

Variable	*n*	%
Sociodemographic Characteristics		
Age, mean ± SD (median; range)	55.1 ± 9.0	(55.0; 37–81)
Sex		
Male	36	45.0
Female	44	55.0
Race/Ethnicity		
Hispanic/Latino	45	56.3
African American	25	31.3
Other (White, Asian)	10	12.5
Employment		
Employed	18	22.5
Unemployed/Homeless	51	63.8
Other (retired, disabled, unknown)	11	13.8
Insurance Status		
Uninsured	34	42.5
Insured	46	57.5
PMAP Enrollment		
No	34	42.5
Yes	46	57.5
BMI Category		
Normal (<25 kg/m^2^)	24	30.0
Overweight (25–30 kg/m^2^)	29	36.3
Obese (>30 kg/m^2^)	27	33.8
Smoking Status		
Non-smoker	40	50.0
Smoker (current or former)	40	50.0
Disease Characteristics		
ISS Stage		
Stage 1	13	16.3
Stage 2	35	43.8
Stage 3	18	22.5
Unstaged/Not Available	14	17.5
Cytogenetic Risk		
Standard risk	44	55.0
High risk	24	30.0
Not available	12	15.0
ECOG Performance Status		
ECOG ≤ 2	66	82.5
ECOG > 2	4	5.0
Not available/not applicable	10	12.5
Bone Disease		
No	11	13.8
Yes	69	86.3
Dialysis Required		
No	71	88.8
Yes	9	11.3
Comorbidities		
Hypertension	28	35.0
Diabetes Mellitus	19	23.8
Hyperlipidemia	13	16.3
Chronic Kidney Disease	17	21.3
Depression	32	40.0
Multiple Myeloma Management		
Induction Regimen		
VRD	56	70.0
KRD	2	2.5
CyBorD	19	23.8
Other	3	3.8
Standard Regimen (VRD or KRD)		
Yes	56	70.0
No	24	30.0
IMiD Agent		
Lenalidomide	76	95.0
Pomalidomide	4	5.0
Transplant Eligibility		
Eligible	29	36.3
Not eligible	43	53.8
No information	8	10.0
Bone-Directed Therapy		
No	16	20.0
Yes	64	80.0
Maintenance IMiD		
No	41	51.3
Yes	39	48.8
Vital Status at Last Follow-up		
Alive	62	77.5
Deceased	18	22.5

PDC = proportion of days covered; PMAP = Patient Medication Assistance Program; BMI = body mass index; ISS = International Staging System; ECOG = Eastern Cooperative Oncology Group;; VRD = bortezomib/lenalidomide/dexamethasone; KRD = carfilzomib/lenalidomide/dexamethasone; CyBorD = cyclophosphamide/bortezomib/dexamethasone; IMiD = immunomodulatory drug. R-ISS staging was not consistently available across the full study period (2010–2022) due to adoption of R-ISS criteria in 2015. Baseline bone marrow plasma cell burden and complete cytogenetic data were not uniformly documented.

**Table 2:** Bivariate analysis of PDC by subgroups (*n* = 80).

Variable	Patients (*n*, %)	Median PDC	*p*-Value
Age (mean, SD)	55.12 ± 9.0	0.25	0.266
Gender			
Male	36 (45.0)	0.33	0.058
Female	44 (55.0)	0.46	
Race/Ethnicity			
African American	25 (31.3)	0.33	0.107
Hispanic/Latino	45 (56.3)	0.46	
Other (White, Asian)	10 (12.5)	0.25	
Employment			
Employed	18 (22.5)	0.33	0.843
Unemployed	51 (63.8)	0.36	
Other	11 (13.8)	0.46	
Insurance			
Uninsured	34 (42.5)	0.38	0.205
Insured	46 (57.5)	0.33	
Prescription Medical Assistance Program (PMAP)			
No	34 (42.5)	0.31	0.029 *
Yes	46 (57.5)	0.40	
Body Mass Index (BMI)			
Normal (19–25)	24 (30.0)	0.35	0.351
Overweight (25–30)	29 (36.3)	0.33	
Obese (>30)	27 (33.8)	0.46	
Smoking Status			
Non-smoker	40 (50)	0.42	0.380
Smoker	40 (50)	0.33	
Transplant Eligible			
No	43 (53.8)	0.46	0.022 *
Yes	29 (36.3)	0.27	
No information	8 (10.0)	0.31	

* *p*-value < 0.05 is statistically significant.

## Data Availability

The data presented in this study are available upon request from the corresponding author. The data are not publicly available due to patient privacy restrictions.

## Abbreviations

IMiD	Immunomodulatory drug
MM	Multiple myeloma
PDC	Proportion of days covered
PMAP	Patient Medication Assistance Program
REMS	Risk Evaluation and Mitigation Strategy
PFS	Progression-free survival
OS	Overall survival
PI	Proteasome inhibitor
SES	Socioeconomic status
BMI	Body mass index
EHR	Electronic health record
FDA	U.S. Food and Drug Administration
